# Improving Efficiency of Direct Pro-Neural Reprogramming: Much-Needed Aid for Neuroregeneration in Spinal Cord Injury

**DOI:** 10.3390/cells12202499

**Published:** 2023-10-20

**Authors:** Daria A. Chudakova, Ekaterina M. Samoilova, Vladimir P. Chekhonin, Vladimir P. Baklaushev

**Affiliations:** 1 Federal Center for Brain and Neurotechnologies, Federal Medical and Biological Agency of Russia, 117513 Moscow, Russia; 2 Engelhardt Institute of Molecular Biology, Russian Academy of Sciences, 119991 Moscow, Russia; 3 Federal Research and Clinical Center of Specialised Medical Care and Medical Technologies FMBA of Russia, 115682 Moscow, Russia; 4 Department of Medical Nanobiotechnology of Medical and Biological Faculty, Pirogov Russian National Research Medical University, Ministry of Health of the Russian Federation, 117997 Moscow, Russia

**Keywords:** directly reprogrammed cells, neural progenitor cells, neural tissue regeneration, central nervous system injury, spinal cord injury, cell fate, cell reprogramming, therapeutic strategies, clinical application

## Abstract

Spinal cord injury (SCI) is a medical condition affecting ~2.5–4 million people worldwide. The conventional therapy for SCI fails to restore the lost spinal cord functions; thus, novel therapies are needed. Recent breakthroughs in stem cell biology and cell reprogramming revolutionized the field. Of them, the use of neural progenitor cells (NPCs) directly reprogrammed from non-neuronal somatic cells without transitioning through a pluripotent state is a particularly attractive strategy. This allows to “scale up” NPCs in vitro and, via their transplantation to the lesion area, partially compensate for the limited regenerative plasticity of the adult spinal cord in humans. As recently demonstrated in non-human primates, implanted NPCs contribute to the functional improvement of the spinal cord after injury, and works in other animal models of SCI also confirm their therapeutic value. However, direct reprogramming still remains a challenge in many aspects; one of them is low efficiency, which prevents it from finding its place in clinics yet. In this review, we describe new insights that recent works brought to the field, such as novel targets (mitochondria, nucleoli, G-quadruplexes, and others), tools, and approaches (mechanotransduction and electrical stimulation) for direct pro-neural reprogramming, including potential ones yet to be tested.

## 1. Introduction

Acute traumatic damage to the central nervous system (CNS) is one of the major health problems continuing to loom large worldwide and affects a significant number of individuals, many of whom die as a result or remain disabled for the rest of their lives. Such damage includes abrupt or sustained traumatic injuries of the spinal cord (traumatic spinal cord injury; SCI), brain (traumatic brain injury; TBI), and peripheral nerves and can be subdivided into primary and secondary injuries, caused by direct structural damage and subsequent molecular and cellular response of the tissue, respectively. It is estimated that approximately 2.5–4 million people are affected by SCI worldwide [1]. SCI bears great socio-economic consequences as it often affects young individuals.

At the same time, as a result of global aging, the incidence of SCI among aging people may increase as well. Despite all advancements made in translational neuroscience, the most effective therapeutic approach to SCI that minimizes damage, regains spinal cord conductivity, and replaces injured non-functioning tissue with fully functional tissue has not been found yet. 

There is a wealth of reviews on the topic of post-SCI neuroregeneration, and the number continue to rise ([2,3,4,5], to name but a few). Many of them focus on the use of transplanted cells as a therapeutic approach to SCI. There are three main types of such cells that are commonly used, namely, induced pluripotent stem cells (iPSCs), multipotent mesenchymal SCs (MSCs) and directly reprogrammed neural progenitor cells (drNPC). Of them, iPSCs pose tumorigenic risks, while MSCs—despite promising results and undoubted clinical potential—do not demonstrate truly breakthrough results in clinical trials for SCI, as discussed in detail further in the text. Thus, using significantly less tumorigenic patient-tissue-derived drNPCs that are capable of fast pro-neuronal differentiation is an attractive alternative strategy. 

Therefore, in our narrative review, we only focus on directly reprogrammed neural cells (drNPCs) and approaches to increase the efficiency of direct pro-neural reprogramming. We leave other therapeutic approaches to SCI, for example, immunotherapy [6], the use of tissue-engineered scaffolds (TES), and animal models of SCI recently reviewed elsewhere beyond the scope of the current review. Our work fills several critical gaps in knowledge, namely, (1) it briefly overviews the most recent literature on the use of reprogrammed cells for SCI, (2) summarizes existing approaches to enhance direct reprogramming to pro-neuronal lineage, and (3) proposes several possible approaches yet to be tested.

## 2. Adult Neurogenesis in CNS Post-SCI 

Lower vertebrates such as the zebrafish are capable of regenerating the injured CNS and even the spinal cord (though to various extents depending on the species) [7], and some other vertebrates, for example, the amphibian axolotl, can also regenerate anatomically dissected spinal cord tissue [8]. Furthermore, in mammals, the possibility of scar-free spinal cord repair was demonstrated in neonatal animals (mice) [9]. However, the functional recovery of adult human CNS tissues after damage is limited by their very low regenerative ability. Furthermore, while, in the case of TBI, neuronal plasticity may allow for the compensating for local damage, SCI generally cannot be repaired or functionally compensated, and the results for debilitating consequences include complete loss of motor function, paralysis (paraplegia or quadriplegia), and dysautonomia. 

Adult neurogenesis in the CNS of laboratory model mammals (mice) is restricted to several regions of the brain and is not sufficient to replace the tissue lost due to neurotrauma [10]. Moreover, modern single-nucleus transcriptomic studies have not confirmed neurogenesis in the neural stem cell niches of human adults at all, based on the absence of robust transcriptomic and histological signatures of neurogenesis [11]. As for SCI, the current concept is that the human spinal cord lacks a capability for neurogenesis, although some works support the view that ependymal cells of the central canal lining may have some neurogenic potential (i.e., the ability to generate neurons) in vitro in some mammals [12]. Also, the presence of cells expressing markers of neuroblasts was reported in the post-SCI lesion site in mice, suggesting the possibility of the cellular shift toward neurogenesis after SCI [13] (notably, according to this work, only spinal NG2 glia cells but not astrocytes or ependymal cells have neurogenic potential). A recent study in mice demonstrated that neurons, following SCI, can revert to a somewhat embryonic-like state (as confirmed by their “regenerative transcriptome” indicating a reversal to an “embryonic transcriptional state”), and such a state can be sustained via grafts of neural progenitor cells (NPCs) [14]—this approach is yet to be evaluated in primates. It should also be noted that molecular and cellular responses to SCI, as well as mechanisms of neurogenesis and neuroregeneration in general, in humans and small laboratory animals (such as commonly used rodents or zebrafish) are fundamentally different in some aspects (as reviewed in [15]). 

The zebrafish is perhaps the most popular animal model worldwide for post-SCI regeneration study [16]. The axolotl is also an attractive model, used not only as a model of spinal cord transection but also as a more clinically relevant blunt contusion injury [17]. As both the zebrafish and axolotl are so-called regenerative species, they are instrumental in studying the mechanisms of post-SCI neuroregeneration that are “dormant” in non-regenerating species and, in this particular field of research, cannot be replaced by non-human primate models. However, once the molecular mechanisms underlying the aforementioned neuroregeneration are deciphered using these models, attempts can be made to apply this knowledge to human SCI regeneration.

Some large animal models of SCI (such as porcine and, especially, non-human primate models [18]) more closely resemble the pathophysiology of human SCI and, therefore, are considered more predictive and attractive intermediary translational models of SCI (as reviewed in [19]), though no model completely recapitulates all the processes occurring during human SCI. 

Thus, despite the wealth of works utilizing animal models of SCI, not all of them may have immediate translational potential or clinical value. Either way, as mentioned, spinal cord damage in humans cannot be fully mitigated through endogenous mechanisms via stem cell differentiation into neuronal lineage.

## 3. Therapeutic Approaches to SCI

SCI is a multi-step disorder. The timeline of SCI and its sequelae can be divided into four phases: immediate (occurs within the first two hours after the trauma), acute or inflammatory (occurs within the first couple of days after the tissue damage, characterized by excitotoxicity, microglia activation, the post-traumatic inflammation and infiltration of the lesion area by immune cells, the imbalance of ionic homeostasis, and the loss of neurons and glial cells due to necrosis or programmed cell death), sub-acute (starts within two weeks after the initial damage, mainly characterized by tissue scarring and axon demyelination caused by the loss of oligodendrocytes), and chronic (takes months and years after the damage, characterized by further scarring, cellular death, demyelination, etc.) (as comprehensively reviewed in [20]). 

It is intuitively clear that depending on the phase of SCI and, therefore, depending on the different molecular, mechanical, and cellular states of the injured tissue micro-environment, curative strategies must also differ. During the first phase, the common therapeutic strategies are surgical decompression, anti-edema therapy, and anti-inflammatory therapy. During the acute period, the most common therapeutic approaches are anti-inflammatory and immunocorrective therapy, as well as strategies aimed to prevent excessive scar formation. During the subacute phase, the transplantation of autologous cells can be performed, and anti-inflammatory therapy, the activation of regeneration, and the prevention of scar tissue proliferation are still relevant. Finally, during the chronic phase, the prevention of ascending and descending axonal degeneration, the stimulation of neurite growth, enhanced rehabilitation with sensory input, and the activation of spinal neural networks are recommended. Some therapeutic interventions are most efficient if started during the first phase of SCI, thus preventing, delaying, or diminishing subsequent adverse ramifications, whereas others are instrumental during the later phases. For example, the inhibition of apoptosis within the first hours after the injury and the implantation of stem/progenitor cells within TES at later stages.

While acknowledging the importance of early interventions, in this review we focus on the latter strategy, including cell-based therapies. 

Briefly, the main purpose of the implantation of stem/progenitor cells to the lesion area post-SCI is to replenish cells lost as a result of trauma or to help the remaining “host” cells repair the damaged tissue.

Historically, common approaches to manage SCI and its consequences included surgery (spinal decompression surgery), physical therapy, and pharmacotherapeutic interventions. However, significant strides have been made. Santiago Ramon y Cajal, the “founding father” of neurobiology, introduced a dogma (as applied to neurons): “Everything may die, nothing may be regenerated”. A recent breakthrough in stem cell (SC) biology, the development of protocols for cell reprogramming, including direct neural reprogramming and the generation of new neurons via in situ cell reprogramming, revolutionized the field [21]. Such an approach was further enforced by single-cell transcriptomics, allowing for the identification of novel targets and delineation of the fine-tuned mechanisms of post-SCI tissue remodeling [22], advances in biomaterial-based tissue repair in SCI (reviewed in [23]), and the development of novel drug delivery systems (DDS) based on nanoparticles for post-SCI regeneration [24]. 

**Figure 1:**
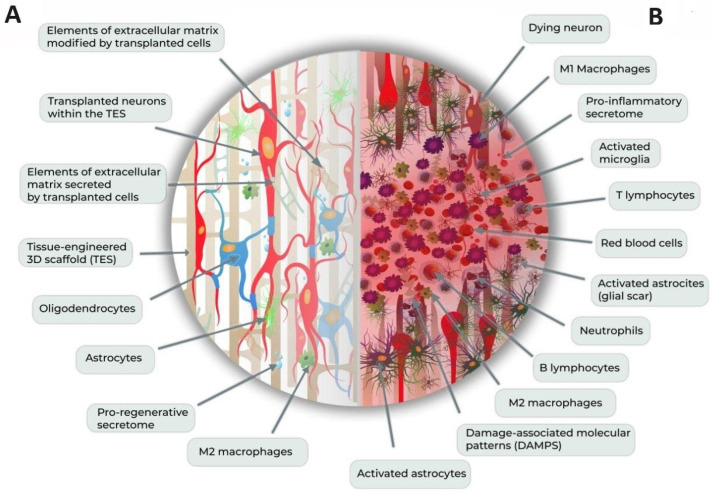
**Combination therapy approaches to SCI. (A) Application of TES and cell therapies to SCI.** TES transplanted to the lesion site provide mechanical support to the tissue, can be used as a drug delivery system, might have conductive properties, and guides cell differentiation, proliferation and migration. NPC cells transplanted within TES might either differentiate toward neurons/oligodendrocytes, thus compensating for the cell loss due to the SCI and/or contribute to neuroregeneration via pro-regenerative and neurogenic secretome milieu, modification of the extracellular matrix facilitating tissue repair, and recruitment or modulation of functionalities of sub-populations essential for post-SCI recovery (for example, M2 macrophages). (**B**) **SCI lesion site.** Without any therapeutic interventions, SCI lesion site is characterized by hemorrhage, edema, pronounced cell death, inflammation, glial and fibrotic scar formation, exacerbated tissue damage, secretion of pro-inflammatory molecules, and recruitment, activation, or phenotype switch of multiple sub-populations of cells (macrophages, residential astroglia, and others).

Currently, cutting-edge approaches to neural regeneration and functional restoration post-SCI are multi-pronged and propose using various combinations of supportive TES with cell therapies (Figure 1), including cell therapy utilizing induced pluripotent stem cells (iPSCs) or directly reprogrammed neural precursor/progenitor cells (drNPCs), electrical epidural stimulation, and the application of bio-active compounds modulating molecular pathways critical for tissue regeneration and its normal functioning [25,26,27]. 

Notably, such TES can also be used as a drug delivery system [28], including conductive scaffolds [29], etc.

Finally, neuromodulation devices for artificial neural connections allowing neural data transmission from one undamaged part of the spinal cord to another might complement the aforementioned curative strategies [30]. 

## 4. Stem Cell Therapy for SCI

Cell transplantation for therapeutic applications has been gaining momentum over the last couple of decades. In the case of SCI, there were attempts to use several types of cells for transplantation therapy—SCs, cord blood cells, olfactory ensheathing cells (OECs), and others [31]. Of particular interest are SCs. Briefly, SCs are, by definition, cells that are capable of self-renewal and have the ability to differentiate into several cell types [32]. Based on their origin, SCs can be subdivided into embryonic SCs, adult or somatic SCs, and induced SCs; and, based on their ability to differentiate, they are subdivided into totipotent, pluripotent, multipotent, and unipotent (here, we refer readers to several recent reviews on this subject, for example, [33]. Regenerative therapy with the use of SCs proved to be a success for many conditions, allowing functional and structural tissue restoration, at least partially. Promising trends were also demonstrated in the case of using SCs for post-SCI therapy [34], including results both from animal studies and from clinical trials [35]. For example, the transplantation of human neural stem cells to the injured spinal cord of primates had some restorative effects; in particular, grafted cells survived for several months, host sinapse-forming axons regenerated into the graft, and implanted cells also extended their axons through the recipient tissues and formed synapses [36].

To sum it up, transplanted SCs may contribute to post-SCI neuronal regeneration via several mechanisms—by replacing dead cells at the lesion site and differentiating to the cells of neuronal lineage, partially restoring disrupted neuronal circuitry, promoting the remyelination of axons and contributing to long-distance axon regeneration, the secreting of neurotrophic factors, anti-inflammatory cytokines, pro-angiogenic factors, exosomes with bio-active cargo, their neuromodulatory activities, the potential for the activation of endogenous neurogenesis, etc.

There are three main strategies to use stem cells for post-SCI tissue regeneration—(1) to stimulate endogenous SCs, (2) to use the SC-derived secretome, or (3) to transplant exogenous SCs into the damaged tissue. 

As for the stimulation of endogenous SCs, it is tempting to agree with the recent statement of DeFrates et al. that an attractive—although challenging at the same time—therapeutic strategy for SCI may be to evoke endogenous regenerative mechanisms in the damaged tissue of so-called “non-regenerative” species such as humans, which is incapable of epimorphosis. For example, one of the key influencers of mammalian tissue regeneration is transcriptional factor hypoxia-inducible factor-1 (HIF-1a), also known to be implicated in stem cell maintenance [37]. Supposedly, deciphering the regulation of molecular networks orchestrated by HIF-1a in regenerating species following injury may help us to evoke the endogenous neuroregenerative potential in non-regenerating species. It should be noted that the stabilization of HIF-1a post-SCI might be beneficial for many other reasons, as it leads to the inhibition of neural apoptosis and enhances axon regeneration [38]. The activation of dormant ependymal cells post-SCI might also be in line with the aforementioned strategy. It is suggested that the major underlying mechanism of spinal cord regeneration in the axolotl is through the Sox2-dependent “awakening” of the dormant ependymal cells post-SCI, although detailed mechanisms are still enigmatic (as reviewed in [39]). In humans of a certain age, the latent ependymal population can be activated by injury, although its stem cell potential is a highly controversial topic, and the possibility of adult neurogenesis in humans (even if induced by external stimuli) remains in doubt. In mice, the population of immature ependymal cells as potential spinal cord stem cells was recently identified [40]. Such approaches, of course, are only speculative, given that the identities of the ependymal cells of the adult spinal cord of the axolotl and humans (and even more closely related to human mice) are different, and there are other fundamental molecular and cellular differences between the species. Nevertheless, deciphering the molecular mechanisms of spinal cord regeneration in so-called “regenerative” species may provide new insight into this subject, and cell-based screenings or an in silico search for the bio-active compounds modulating such activation might be of potential clinical value.

As for the SC-derived secretome, based on animal studies SC-derived exosomes and their molecular cargo have the potential to become a cell-free therapy for SCI [41]. Apart from exosomes, other types of bio-active molecules within the SCs’ secretome might also have therapeutic value (as reviewed in [42]), and their intravenous administration might become one of the treatment strategies for SCI.

As for the third strategy based on the transplantation of exogenous SCs, there is mounting experimental evidence supporting the anti-inflammatory role of MSCs in post-SCI treatment and their ability to stimulate nerve regenerative signaling pathways and promote vascular repair [43]. The strategy of cell-based therapy for SCI became particularly attractive after the publication of the seminal work of Takahashi and Yamanaka, describing the approach to reprogram somatic cells into a pluripotent state via forced expression of several transcriptional factors (TFs) [44]; this work led to a “gold rush”-like era of extensive efforts in SC-based therapy research and development. Since then, significant progress in the management of neurotrauma and SCI in particular was achieved by applying SCs as a therapeutic tool, including not only widely used MSCs but also the SC-derived biologically active secretome, induced pluripotent stem cells (iPSCs), and directly reprogrammed multipotent neural stem cells (drNSCs) or neural progenitor cells (drNPCs) capable of giving rise to neurons, astrocytes, and oligodendrocytes [45,46,47]. A detailed comparison of the several types of SCs in the context of SCI cell therapy can be found in a recent encyclopedic review by Shao A. et al. [48], and, hereafter, we focus on drNPCs.

## 5. Directly Reprogrammed Pro-Neuronal Cells

From the point of view of a clinical neurologist, the use of drNSCs/drNPCs compared to iPSCs or MSCs has some advantages. First and foremost, it is widely accepted that, in the case of using directly reprogrammed cells, the tumorigenic risk is significantly lower compared to that of iPSCs. Secondly, the procedure of direct reprogramming is much faster and cheaper compared to iPSC generation. Thirdly, direct reprogramming preserves the epigenetic profile of the cell, which is impossible in the case of iPSCs [49,50]. Many clinical trials demonstrated some benefits of MSC in SCI, perhaps due to the paracrine effect of the MSC-derived secretome. However, despite encouraging results with MSCs, drNPCs are even more promising as a candidate for the cell-based therapy of SCI, given their “pro-neuronal” features. Admittedly, in the case of in vitro direct reprogramming, the population of reprogrammed cells has some degree of heterogeneity; thus, there is always a risk of the transplantation of the sub-population of non-converted non-neuronal cells. It might be assumed that the transplantation of unmodified non-neuronal cells to the lesion site post-SCI (for example, fibroblasts, most commonly used for generation of drNPC) is either not detrimental, does not lead to any functional improvement [51], or perhaps might contribute to fibrotic scar formation [52]. Thus, transplantation of the mixture of cells, some of them being non-neuronal cells, might potentially pose some risks. The commonly used strategy to mitigate this issue is the marker-based selection of cells from the heterogeneous population before implantation (for example, cells are captured by specific antibodies conjugated to magnetic beads, and fluorescence-activated cell sorting or magnetic-activated cell sorting are used [53]). This risk is also mitigated in case of the in situ reprogramming of cells already present in the lesion region. Furthermore, there were several attempts to perform direct reprogramming in situ, suggesting the potential feasibility of such an approach. Of course, any genetic manipulation involving exogenic integration bears potential risks; thus, the transplantation of non-modified cells like MSCs seems to be safer than genetically manipulated iPSCs or drNPCs (assuming that they were generated via the ectopic expression of TFs delivered by viral constructs). Either way, the SC-derived secretome (MSC-, iPSC-, or drNPC-derived) can be used as a safer cell-free therapeutic agent, given its role in pro-regenerative paracrine signaling. For example, as we previously demonstrated in non-human primates, drNPC transplantation partially compensates for the limited regenerative plasticity of the adult spinal cord and contributes to its functional improvement post-SCI (as can be assessed by the commonly used functional tests, namely, the kinematic assay, neurological assessment, and the neurophysiological investigation of the evoked potentials (SSEP and MEP)), supposedly through paracrine trophic support in the areas of active growth cone formation [54]. 

Hitherto, a wealth of protocols was established for the generation of iPSCs from somatic cells of any lineage and their subsequent differentiation into the cells of neuronal lineage, for different species including humans. In parallel, a mammoth worldwide effort was put into developing protocols for direct neuronal reprogramming, allowing for the direct generation of different types of cells of neuronal lineage from fully differentiated non-neuronal cells but avoiding the pluripotent state (as summarized in our recent review [55] and by others [56]; Table 1).

A number of studies during the last decades have accumulated evidence demonstrating the potential clinical value of NPCs, both non-differentiated and differentiated, including differentiation in region-specific cells. To name but a few, in a recent work by Xu et al., collagen scaffolds were populated in vitro by human NPCs, which were induced to differentiate into different types of dorsal and ventral neuronal cells. Next, scaffolds were transplanted into animal models of SCI (mice and rhesus monkeys) and demonstrated therapeutic effects that were more prominent compared to the implantation of non-differentiated NPCs within the same type of scaffolds [57]. Furthermore, the direct reprogramming of human astrocytes into early neuroectodermal cells and their subsequent implantation into the SCI lesion area in a mouse model resulted in the differentiation of the implanted cells into region-specific neurons that formed synapses with the neurons of the host [58]. 

Mounting evidence from other similar studies suggests the potential clinical value of such an approach and advocates for the development and optimization of reprogramming methods. 

**Table 1:** Current methodological approaches to direct neuronal reprogramming.

Method/Molecular Tool ^(selected examples)^	Species	Cells of Origin/ Target Cells	Type of Study	Selected Representative Reference
Overexpression of TFs * ^(Neurogenin 2, Pax6, Sox2, Sox11)^	human	bone marrow MSC/NPCs	in vitro, using non-integrating plasmids	[59]
	human	fibroblasts/motor neurons	in vitro, using integrating viral vectors	[60]
	human	adult dermal fibroblasts/NPCs	in vitro, using synthetic mRNA (or synthetic modified mRNA) encoding reprogramming TFs	[61]
Silencing/overexpression of TFs * ^(p53/Oct3/4, Sox2, Klf4, L-myc, Lin28;^ ^REST/Ascl1, Brn2)^	human	adult dermal fibroblasts/neurons	in vitro, using non-integrating plasmids	[62]
	human	adult dermal fibroblasts/neurons	in vitro, in combination with RNA interference	[63]
Small molecules ^(Valproic acid, Chir99021, Repsox, forskolin, i-Bet151, ISX−9; Pax6, Sox2)^	human	astrocytes/neurons	in vitro	[64]
Small molecules and microRNAs ^(Valproic acid, mir−302/367)^	human	astrocytes/neuroblasts	in vitro and in vivo	[65]
Small molecules and TFs/regulatory proteins ^(suppression of p53, overexpression of Oct4, Chir99021, Repsox, SB431542, Y27632)^	human	astrocytes/neurons specific to the dorsal and ventral domains in vitro	in vitro	[58]
MicroRNA(s) and TFs ^(miR−9/9*−124, BCL11B, DLX1, DLX2, MYT1L)^	human	fibroblasts/striatal neurons	in vitro	[66]
CRISPR activators ^(activators of Brn2, Ascl1, Myt1l)^	mice	MEFs/neurons	in vitro	[67]
Overexpression of TFs ^(NeuroD1)^	mice	astrocytes/neurons in situ	in situ, adeno-associated virus and retrovirus-based systems	[68]
Overexpression of TFs ^(Sox2)^	mice	N2 glia/neurons	in situ, integrating viral vectors	[69]
RNA interference ^(silencing of PTB)^	mice	astrocytes/DA neurons	in situ, shRNA-encoding construct	[69]
RNA interference/small molecules ^(silencing of NOTCH/DAPT)^	mice	astrocytes/neurons	in situ, shRNA-encoding construct	[70]

* in different combinations, in combination with optional addition of small molecules, overexpression of regulatory proteins, and use of other molecular tools.

Briefly, in the majority of such reprogramming protocols, several key TFs, the so-called pioneer TFs—a subset of TFs that are capable to bind “silent” (“closed”) chromatin and recruit other TFs to initiate lineage-specific transcription programs—are ectopically expressed in cells, resulting in global and local changes in the epigenome, transcriptome, metabolome, etc., as well as the overall shift of the cell fate.

Such TFs might be optionally supplemented or substituted by particular small molecule inhibitors/activators or regulatory microRNAs, proteins, etc., as well as supplemented by optional so-called cooperative TFs. In the case of direct reprogramming, there are also protocols that might require the repression of some lineage-specific TFs or “barrier” factors to achieve cell fate conversion (for example, REST1 is one of such “barrier” factors, and its suppression mediates the conversion of fibroblasts to neuronal lineage [63]). 

There are different systems that can be used for TFs’ ectopic expression, for example, the delivery of non-integrating plasmids via electroporation or lipofection, the delivery of genetic material with the aid of lentiviral vectors transduction, the use of Sendai virus (SeV) vectors, and others. There are advantages and disadvantages of different systems, as discussed elsewhere. Although many protocols claim that it is possible to generate drNPCs only using chemical reprogramming agents, without any TFs, we found that in many cases such “reprogramming” by fibroblasts allows to generate cells expressing some markers of the target cells of neuronal lineage (for example, beta-III-tubulin, commonly and most exclusively found in neurons) and also resembling the target cells in terms of size/shape, but these cells also retain some markers of fibroblasts and may fail in functional tests. One of the possible explanations of such an inconsistency in the published data is the fact that, in many publications reporting protocols for reprogramming, there were no functional tests performed; or, maybe, direct reprogramming via chemical cocktails should only be performed under hypoxic conditions, as reported in the original work by Cheng et al., who introduced this methodology [71]. We insist that functional tests (for example, the commonly used electrophysiology tests, such as whole-cell patch clamp recordings [59]), are necessary, because the presence of some neuronal markers does not guarantee that the cell is a functional neuron.

Overall, even though small molecules are undoubtedly very attractive as clinical tools, perhaps using TFs (alone or in combination with other factors) is still the best strategy for direct pro-neuronal reprogramming; thus, using direct reprogramming for SCI therapy remains a knotty problem, given the hurdles of ectopic expression of reprogramming TFs in situ and the overall risks of genetic intervention (such as forced expression of TFs) as a therapeutic tool. This also raises a burning question: what evidence is necessary and sufficient to rigorously and convincingly confirm the direct reprogramming of cells to neuronal lineage? It should be noted that, apart from chemical reprogramming, other methods for the direct reprogramming (without the use of TFs) of somatic cells into neuronal lineage also exist, for example, via the transcriptional and chromatin modulations by the CRISPR activator system [67]. Finally, there are non-chemical and non-genetic routes to reprogram fibroblasts to pro-neuronal cells, for example, based on mechanotransduction signaling and biophysical stimuli, as discussed in detail further in the text. 

There are also several reports about the possibility of the in situ direct conversion of non-neuronal somatic cells to neurons, including in the case of the post-SCI tissue micro-environment. Firstly, there is a claim that the conversion of astrocytes was achieved in situ post-SCI via reprogramming by NeuroD1 in mice [68]. Secondly, the in situ reprogramming of NG2 glia toward a neurogenic state in mice was reported [69]. Lastly, it was suggested in recent work that the pharmacological inhibition of NOTCH1 signaling can also trigger the direct conversion of astrocytes to neurons in situ post-SCI in mice (the pro-neuronal conversion was assumed based on observed changes in expression of pro-neural TFs, namely, NeuroD1, NeuroD2, Pax6, Lmx1a, and Lhx6) [70]. However, the presumed possibility of the direct conversion to neurons in situ remains a point of controversy, given the possibility of the misinterpretation of the observed results or the flows of the molecular tools used in the aforementioned studies [72,73]. 

## 6. Strategies to Increase Efficiency of Direct Reprogramming to Neuronal Lineage

**Figure 2:**
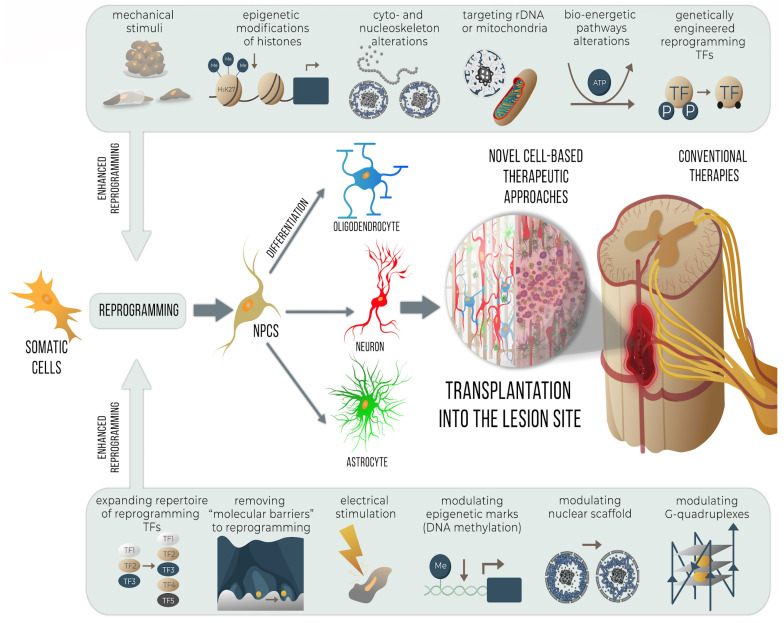
**Schematic representation of approaches to improve the efficiency of direct pro-neural reprogramming.** Reprogramming efficiency can be enhanced via modulation of mechanotransduction and use of particular mechanical stimuli; modulation of epigenetic barriers to reprogramming (DNA methylation and/or histone modifications, globally or at particular loci); modulating chromatin organization, nuclear scaffold, and cytoskeleton; targeting rDNA and nucleoli; altering bioenergetic pathways and mitochondria; expanding the repertoire of reprogramming TFs and usage of genetically engineered ones; removing “molecular barriers” to reprogramming (silencing of particular TFs or regulatory proteins); electrical stimulation of cells; targeting G-quadruplexes or other regulatory DNA/RNA structures. Applied in vitro at the stage of direct reprogramming of non-neuronal somatic cells toward the pro-neural cell fate, they precede the transplantation of reprogrammed cells within TES to the SCI lesion site, as a novel cell-based therapy complementing conventional therapies.

The low efficiency of pro-neuronal reprogramming is a hurdle to its clinical application [74]. Thus, methodological approaches allowing to increase the efficiency/speed of direct reprogramming to neuronal lineage are constantly being developed. Indeed, any molecular, mechanical, or physical manipulations “paving the way” for the preferential and easier conversion of the cell into the particular target cell type facilitate reprogramming (Figure 2). 

Perhaps it might be manipulations affecting the cyto- and nucleoskeleton. As a proof of principle, targeting the actomyosin contraction of the cytoskeleton in fibroblasts evoked an “intermediate” neuron-like state in cells, making them more prone to subsequent reprogramming into neurons [75]. Based on RNA-seq data, Herdy et al. uncovered several pathways critical for the conversion of fibroblasts to neurons (for example, integrin signaling, HIF-1a signaling, Rho-family GTP-ase signaling, and others) and identified molecular modulators of these pathways, allowing for a significant increase in the yield of reprogrammed cells [76]. In particular, among these “reprogramming booster” molecules were Pyrintegrin (Integrin activator) and ZM336372 (Raf-1 activator); potentially, both compounds could promote cytoskeleton reorganization, thus minimizing mechanical stress-induced apoptosis during cell fate conversion. 

A very interesting finding was reported in the recent article by Yang J. et al. Using mouse fibroblasts, the authors introduced DNA double-strand breaks in the region encoding ribosomal RNA (the rDNA region) in nucleoli, allowing them to be faithfully repaired, and observed that the cell fate of the treated cells was “primed” toward neurons. In particular, they observed changes in the histone modifications (a decrease in the H3K27me3 mark) in the promoter regions of the genes *Neurod1* and *Nef*h, which play key roles in determining “neuronal fate”, as well as transcriptome changes in the gene ontologies’ neuronal processes. In such “primed” fibroblasts, direct reprogramming with chemical agents was more efficient compared to with non-primed fibroblasts, as assessed by the derepression of *Neurod1* and *Nefh* and the neuron count [77]. This finding is in line with the previously reported observation that, speaking about long-range chromosomal interactions, rDNA constitutively interacts with regions related to nervous system development and may play a role in the regulation of their transcriptional activity [78]. Moreover, the direct interaction of rDNA with genes involved in differentiation was reported [79], and the role of nucleoli in orchestrating cell fate is well-documented. Finally, during the differentiation of ESCs into NPCs, neural genes located in the regions interacting with rDNA move away from nucleoli to become derepressed [80]. Based on all of the above, we propose that rDNA/nucleoli might be a novel target to “prime” fibroblasts toward direct reprogramming into neurons, which requires further validation.

Furthermore, any shifts in cell fate are associated with—and at least partially initiated by—changes in the epigenome (histone modifications, 3D chromatin organization, and DNA methylation) or, as aptly asserted, “epigenetics: <are> judge, jury and executioner of stem cell fate” [81]. Thus, changes in histone modifications and chromosome long-range interactions might also prime cells toward pro-neuronal reprogramming. Other ways to increase the efficiency of direct reprogramming include so-called “epigenetic resetting” [82] via introducing changes in histone modifications or in DNA methylation. 

For example, the temporarily inhibition of histone deacetylase and bromodomain enhanced the kinetics of neuronal reprogramming of adult fibroblasts in a recent study [83]. Another work reported that the “epigenetic resetting” of fibroblasts by DNA demethylation (treatment with 5-azacytidine), followed by a culture in neuronal differentiating media, resulted in the upregulation of “stemness” genes (*Sox2*, *Klf4*, *Nanog*, and *Oct4*) after the demethylation and expression of neuronal lineage markers after differentiation [84]; however, a significant weakness of this study was the absence of functional tests of the supposedly reprogrammed cells.

Apart from chemical and biochemical cues, physical cues may also affect cell fate. Epigenetic changes leading to a change in the chromatin landscape and, subsequently, transcriptome changes are involved in cell fate regulation, and mechanical stimuli (the stiffness of extracellular matrix, various external stimuli, etc.) can be transmitted through the cytoskeleton to the nucleoskeleton to elicit such epigenetic changes. 

Reorganization of the cytoskeleton and, subsequently, reorganization of the nucleoskeleton architecture and epigenetic modulation can also be achieved via magnetic stimuli. The impact of such cues on neurogenic differentiation was recently comprehensively reviewed [85]. Notably, non-invasive repetitive trans-spinal magnetic stimulation (rTSMS) can also modulate lesion scarring post-SCI in mice, by inhibiting demyelination and enhancing neuronal survival and axonal regrowth in part via stimulating ependymal cells to differentiate into astrocytes and oligodendrocytes [86] (of course, given the fundamental differences of neuroregeneration in mice and humans, the translational significance of such an observation is yet to be assessed). 

As for mechanotransduction, it is known that scaffold-free 3D culture conditions enhance cell stemness, at least for some types of cells, via a variety of molecular mechanisms. As early as 2013, it was claimed that it is possible to convert fibroblasts into NPCs-like cells by forced growth in 3D spheres [87]. Recently, it was demonstrated that culturing astrocytes in a non-adhesive 3D spherical culture system results in the partial conversion of astrocytes into NPC-like cells, as assessed by the levels of expression of the *Sox2*, *Pax6*, *Oct4*, *Nanog*, *Sox10*, and *Pax3* genes [88]. Perhaps such pre-conditioning might make cells more prone to pro-neural reprogramming. Mechanotransduction and the impact of mechanical forces also play a role in cell reprogramming, and the recent thought-provoking study by Song et al. demonstrated that transient nuclear deformation can boost reprogramming efficiency (the conversion of fibroblasts into neurons) via the induction of expression of Ascl1, a bona fide pro-neuronal pioneering TF, and through other mechanisms. Reprogramming was confirmed by elevated levels of neuron-specific markers such as class III beta-tubulin and Tubb3 at early stages of reprogramming, and markers of mature neurons, microtubule associated protein 2 (MAP2), and synapsin at later stages [89]. Somewhat contradictory to the work by Song et al., it was also reported that soft substrates facilitate the direct chemical reprogramming of fibroblasts into neurons [90]. Additionally, the enhanced conversion of fibroblasts to neurons was achieved using tunable electrical stimulation (ES) [91]. There are scarcely any publications on the role of ES in direct pro-neural reprogramming. Moreover, the exact molecular mechanism explaining the impact of ES in pro-neuronal differentiation is still largely unknown. There were several works about the impact of ES on the cell fate of neural stem cells [92] and others published almost a decade ago, as summarized in [93]. Recent work on iPSCs also demonstrated that ES induces robust neuronal fate determination [94]. At the same time, other studies demonstrated that ES stimulates non-neuronal reprogramming as well; for example, it induces the direct reprogramming of human dermal fibroblasts into hyaline chondrogenic cells [95]. As for the direct reprogramming of non-neuronal cells into neuronal progenitors, the role of ES in this process and its exact molecular mechanisms are yet to be deciphered.

Furthermore, manipulations affecting mitochondria can also play a role in the pro-neuronal cell fate switch, given the critical dependence of neurons on mitochondria function [96], the role of mitochondria-mediated metabolic changes in the regulation of neural differentiation [97], and the differences between the mitochondrial proteomes of neurons and other cells. For example, it was shown that the induction of neuron-enriched mitochondrial proteins stimulates direct glia-to-neuron conversion [98] and that increased mitochondrial activity accelerates neuronal differentiation [99]. 

It Is known that clear metabolic differences exist among fibroblasts, NSCs/NPCs, and fully differentiated neurons, in terms of their predominant modes of energy production. During neuronal differentiation, NSCs undergo massive changes in metabolism, including increased OXPHOS [99]; changes of a similar nature if not a similar scale might take place in the case of the direct and indirect reprogramming of fibroblasts to NSCs/NPCs. The inhibition or stimulation of glycolysis decreases or enhances, respectively, the efficiency of iPSC generation from differentiated somatic cells [100].

Nowadays, it is assumed that mitochondria and energy metabolism play a starring—or even controlling—role in both neurogenesis and cell fate regulation [99,101]. Thus, it is not unreasonable to suggest that fibroblasts can be “primed” toward direct reprogramming to neuronal lineage via induced alterations of the major cellular bioenergetic pathways, glycolysis, and oxidative phosphorylation (OXPHOS). Such alterations can be induced, for example, via simple changes in the composition of the growth media [102]. The proposed approach is indirectly supported by the recent publication reporting that the glycolytic switch occurs during the direct reprogramming of fibroblasts to endothelial cells, and such reprogramming can be abrogated via the inhibition of the aforementioned switch [103]. Furthermore, the inhibition of HIF-1a signaling with compound KC7F2 to promote oxidative OXPHOS over glycolysis resulted in the facilitated conversion of fibroblasts to neurons [76]. Notably, metabolic alterations toward the glycolytic metabotype also occur at the stage of blastema assembly and are necessary for cell fate transition [104], highlighting their role in regeneration. Having said this, the bioenergetic of direct reprogramming and the effects of metabolic manipulation on the efficiency and molecular mechanisms of reprogramming remain largely understudied.

A very recent finding is the discovery that TFs ATF7IP, JUNB, SP7, and ZNF207 oppose the cell fate switch in all tested types of cells in mice. They pose a barrier to reprogramming through the downregulation of the genes required for such a switch and by maintaining, in a closed state, the chromatin loci that can be targeted by reprogramming TFs [105]. Perhaps the pharmacological targeting of these TFs might be instrumental in enhancing the capabilities of direct reprogramming. Finding human-specific TFs with similar functions is a task of obvious priority.

Similarly, knockdown of transcription-coupled histone chaperone FACT (resulting in “disorganized chromatin”) in combination with forced expression of TFs known to induce the reprogramming of fibroblasts into neurons increased the reprogramming rate up to 1.5-fold, and, furthermore, the reprogrammed cells were either generated earlier or matured faster [106].

Another guardian of the cell’s fate is the nuclear scaffold and its key components—nuclear lamins—in particular. Indeed, the 3D organization of the genome and, subsequently, the epigenetic state and transcriptional activity of the genes involved in cell fate decisions at least partially depends on the interaction of chromatin with lamins and the overall nuclear (and genomic) morphology. Moreover, in human fibroblast manipulations that affect nuclear scaffolds (such as the transient loss of the core component of the nuclear scaffold, Lamin A/C) resulted in the opening of the previously closed chromatin domains and, thus, facilitated the cellular reprogramming to pluripotency [107]. 

Additionally, the modification of TFs used for cell fate conversion is another promising approach to increase the efficiency of cell reprogramming. For example, Ascl1 is one of the transcriptional regulators determining neuronal differentiation, and its transcriptional activity and capability to drive ectopic neurogenesis is modulated by the multi-site phosphorylation status at serine–proline sites [108]. Thus, it is not surprising that Ascl1 cannot be phosphorylated because its phosphorylation sites are mutated, which causes the enhanced neuronal conversion of astrocytes in mice [109]. Perhaps using such “improved” versions of Ascl1 and other TFs commonly used for cell reprogramming might be an approach to facilitate the cell fate switch in humans as well.

Expanding the repertoire of such TFs is also worth a try. Of particular interest is the recently developed computational tool TRANSDIRE for the prediction of TFs that might induce direct reprogramming in several human cell types (in other words, pioneering TFs that are novel and potentially more potent in terms of evoking the translational changes prerequisite for the phenotype switch toward a particular cell type) [110]. This tool allowed the authors to predict the TFs that could induce direct reprogramming from fibroblasts, based on the combined “omics” data. For neural reprogramming, such novel candidates were MEIS2, ARNT2, PEG3, and others, predicted by the TRANSDIRE alongside known TFs (NEUROD1, REST, and others).

G-quadruplexes (G4s)—four-stranded nucleic acid secondary structures formed by stacked tetrads of guanosine bases in both DNA and RNA—might also play a role in cell fate regulation. In human DNA, they are predominantly formed in enhancers, promoters, and intron/exon borders. G4s are known to be involved in the regulation of transcription, mRNA processing, and localization, including in the case of “neural” genes, and supposedly play a role in cellular differentiation [111,112]. G4s are present in high numbers in human ESCs, and their levels dramatically decrease following differentiation and cell lineage specification; they are also found in SC regulatory elements [113]. It was shown that targeting G4’s stability in NSC promotes the production of oligodendrocyte progenitors [114]. Thus, the role of G4 in generation of drNPCs and their differentiation and the role of the modulators of their stability in the aforementioned processes should be further elucidated.

Next, it was demonstrated in the seminal work by Roy et al., in 2018, that differentiated fibroblasts, if cultured in laterally confined conditions, become less differentiated (SC-like) even in the absence of exogenous reprogramming TFs (the phenomenon of mechanical reprogramming) [115], perhaps through Lef1 activation [116]. A similar approach can also be used to facilitate the cell fate switch, for example, if differentiated somatic cells acquire phenotypic plasticity through the aforementioned approach, which, supposedly, would make them more prone to reprogramming.

Finally, as discussed above, the mechanical, physical, and topological characteristics of the substrate for cell culture dramatically affect some aspects of cell behavior, including the stemness/differentiation potential. This brings to our discussion another tool of post-SCI neuroregenerative therapy—TES. Indeed, in the past few years there seems to have been a steady increase in reports focusing on TES for successful neuroregeneration [117], including SC incorporating TES for SCI treatment (as comprehensively reviewed in [27]). Such TES should meet several requirements: briefly, they have to be biodegradable (allowing for their substitution with tissue), bio-compatible, and bio-mimetic, i.e., recapitulating key properties of the neural tissue, for example, conductivity [29]. In tissues, the extracellular matrix (ECM) is critically important for the spatiotemporal positioning of regulatory biomolecules, for guiding cell migration and growth, and so on and so forth. When transplanted to the lesion site or to the perilesional area, TES should mimic the characteristics of ECM (to some degree) and compensate for its loss. Apart from simple mechanistic compensation for the tissue loss, TES also play many other roles in post-SCI neuroregeneration, as discussed below. It was shown that characteristics of the substrate, in particular surface topography, may guide SCs toward a particular lineage. For example, in the pioneering work of Ghazali et al., adipose-derived SCs were forced toward neural differentiation with the use of cell-imprinted substrates. Briefly, the authors used polydimethylsiloxane silicone substrates to capture and recapitulate the topology of the target human NPCs and, subsequently, cultured adipose-derived stem cells on these substrates, which led to changes in the cell morphology and the upregulation of several markers of neural SCs as well as early neuronal markers [118]. Later, another work confirmed this observation: that cell-imprinted patterns may harness SCs toward a particular cell fate [119]. The sustained delivery of neurotrophic factors (brain-derived neurotrophic factor (BDNF), neurotrophin-3 (NT-3), nerve growth factor (NGF), and others) is another approach to SCI management (as reviewed in [120]). Although the concept of using them for post-SCI neuroregeneration is not novel, there were several recent studies in which they are used in combination with TES as modulators of the differentiation of NSCs, including works in which such factors are immobilized in TES and maintain their neurotrophic functions. For example, in a rat model of SCI, NGF was immobilized in TES based on silk protein nanofiber hydrogels, and it was demonstrated that such an NGF retained its ability to modulate the differentiation of NSCs [121]. Many other up-to-date neuroprotective bio-active molecules for post-SCI therapy were summarized in the recent review by Shah et al. [122].

It should be noted that the differentiation of NPCs toward oligodendrocytes is also being extensively investigated [123,124] and has potential therapeutic applications. However, we leave this topic as beyond the scope of our focused review.

## 7. Potential Obstacles to Be Resolved 

While acknowledging the undoubted translational potential of SCs, here we concede that, in their current state, protocols involving SCs are still not a therapeutic “holy Grail” for SCI; for example, multiple human clinical trials on the use of MSCs in SCI showed results that were definitely encouraging but still not a breakthrough [125]. The clinical value of drNPCs and the drNPC-derived secretome is yet to be comprehensively assessed. The non-negligible hurdles of the cell-based therapeutic approach to SCI therapy are the relatively low survival of the progenitor cells transplanted to the damaged post-SCI tissue and their differentiation toward astroglia in the inflamed tissue micro-environment. Indeed, while some authors provided evidence of the pro-neuronal differentiation and differentiation toward oligodendrocytes of the NPCs in the post-SCI lesion, some authors insisted that transplanted NPCs tend to differentiate toward astroglia (summarised in [126]). As for the survival of the transplanted NPC cells in the post-SCI lesion, in some experiments it was estimated as ∼25% [126]. This can be mitigated by transplanting cells to the lesion site within TES in combination with bio-active molecules modulating cell fate and survival. 

Finally, there are many fundamental differences between conditions in vitro and in vivo, obviously, especially in the case of the post-SCI in vivo micro-environment. For this reason, not all approaches to enhance the efficiency of direct reprogramming in vitro are applicable to in situ cell fate conversion. The protocols for reprogramming and differentiation developed in vitro under normoxia conditions might be not compatible with physioxia conditions. Indeed, normoxia (an atmospheric concentration of O2 ~20–21% commonly used in cell culture experiments) is significantly higher compared to the ~1–11% O2 observed in vivo in tissues (physioxia) [127]. The same can be said about the substrate/micro-environment for cell attachment in vitro and in vivo, the “inflamed” micro-environment of the post-SCI tissue, and so on and so forth. 

Furthermore, several approaches to SCI therapy failed on the path to translation [128]. Noteworthy, the cell-based therapy described in the aforementioned review failed in translation from animal models to clinical use. As for the failure to translate from in vitro to in vivo, several classical examples of the poor predictability of in vitro models come from the field of the preclinical development of CNS-targeted therapies [129]. Thus, unless the linchpin characteristics of the in vivo post-SCI conditions are recapitulated in the culture system used to establish the protocols of cell reprogramming and subsequent differentiation, there are risks that their clinical applicability is limited.

## 8. Conclusions

Directly reprogrammed pro-neuronal cells hold clinical potential, whether used as a standalone therapy or in combination with other therapeutic tools such as TES, small molecules, and others. These cells can also serve as a source of biologically active, pro-regenerative secretomes. Enhancing the efficacy of direct pro-neuronal cell fate conversion could further bolster the translational applications of drNPCs, including their use in SCI neuroregeneration. In this concise review, we provided an overview of various strategies to meet this challenge, including some that have yet to be tested. Encouraging data, including our own, support the continued investigation of neuronal progenitor cells for SCI treatment. 

## Data Availability

Not applicable.
